# Safety pharmacology of acute LSD administration in healthy subjects

**DOI:** 10.1007/s00213-021-05978-6

**Published:** 2021-09-13

**Authors:** Friederike Holze, Toya V. Caluori, Patrick Vizeli, Matthias E. Liechti

**Affiliations:** 1 Division of Clinical Pharmacology and Toxicology, Department of Biomedicine and Department of Clinical Research, University Hospital Basel and University of Basel, Schanzenstrasse 55, CH-4056 Basel, Switzerland; 2 Department of Pharmaceutical Sciences, University of Basel, Basel, Switzerland

**Keywords:** LSD, Safety, Flashback, Blood pressure, Heart rate, Subjective effects, Concentration

## Abstract

**Rationale:**

Lysergic acid diethylamide (LSD) is used in psychiatric and psychological research and investigated as a potential treatment for medical and psychiatric disorders, including depression, anxiety, and cluster headache.

**Objectives:**

Safety data on clinical safety are available from small studies but not from larger samples. We report safety pharmacology data from a large pooled study sample on acute effects of LSD in healthy subjects.

**Methods:**

We conducted a pooled analysis of four double-blind, randomized, placebo-controlled, crossover studies that included a total of 83 healthy subjects and 131 single-dose administrations of LSD. LSD administrations were matched to dose groups according to measured LSD peak plasma concentrations to adjust for uncertainties in the correct LSD dose in some studies. Single doses were 25, 50, 100, and 200 µg of LSD base. We investigated subjective effects (self-rated any drug effect, good drug effect, bad drug effect, and anxiety), blood pressure, heart rate, body temperature, duration of the acute LSD response, acute (12 h) and subacute (24 h) adverse effects, reports of flashbacks, and liver and kidney function before and after the studies.

**Results:**

LSD dose-dependently increased subjective, physiologic, and adverse effects. The dose–response curves for the proportions of subjects with a certain amount of a subjective effect were steeper and reached a higher maximum for positive acute subjective effects compared with negative acute subjective effects. Maximal ratings of > 50% good drug effects were reached in 37%, 91%, 96%, and 91% of the LSD administrations at 25, 50, 100, and 200 µg. Maximal ratings of > 50% bad drug effects were reached in 0%, 9%, 27%, 31% at 25, 50, 100, and 200 µg, respectively. Mean ratings of Oceanic Boundlessness were 10%, 25%, 41%, and 44%, and mean ratings of Anxious Ego-Dissolution were 3.4%, 13%, 20%, and 22% at 25, 50, 100, and 200 µg, respectively. The physiologic effects of LSD were moderate. None of the subjects had systolic blood pressure > 180 mmHg at any time. Peak heart rate > 100 beats/min was observed in 0%, 6%, 20%, and 25% of the subjects at 25, 50, 100, and 200 µg, respectively. Maximal heart rates of 129 and 121 beats/min were observed in one subject at the 50 and 200 µg doses, respectively. Peak body temperature > 38° was observed in 0%, 11%, 7%, and 34% at 25, 50, 100, and 200 µg, respectively. Mean acute adverse effect scores on the List of Complaints were 5.6, 9.2, 12, and 13 at 25, 50, 100, and 200 µg, respectively. Kidney and liver function parameters were unaltered. Six subjects reported transient flashback phenomena.

**Conclusions:**

The single-dose administration of LSD is safe in regard to acute psychological and physical harm in healthy subjects in a controlled research setting.

## Introduction

Lysergic acid diethylamide (LSD) is used recreationally and currently under investigation as LSD-assisted psychotherapy for such indications as depression and anxiety (clinicaltrials.gov ID: NCT03866252, NCT03153579) and as a treatment for cluster headache (NCT03781128). The future medical use of LSD will depend on its safety and efficacy in specific disorders. Modern study data on the safety and efficacy of LSD in patients is lacking. Phase 2 trials are currently being conducted in patients, but more information on clinical safety is needed. Although recent Phase 2 trials with different psychedelics showed only mild and transient adverse events (Andersen et al. 2021), several psychological safety issues have previously been mentioned, including acute anxiety, acute suicidality, and hallucinogen persisting perception disorder (also called “flashbacks”; (Passie et al. 2008; Halpern et al. 2016; Holland and Passie 2011). With regard to safety, the importance of “set and setting” has been highlighted (Johnson et al. 2008), indicating that physiological safety aspects of psychedelics must be considered in clinical settings, in addition to the personality and mental state of the participant and environment where the psychedelic is administered (Barrett et al. 2017; Johnson et al. 2008). Cardiovascular stimulation has also been reported in studies in both healthy participants and patients (Passie et al. 2008; Holze et al. 2021b, 2020; Schmid et al. 2015; Dolder et al. 2015). However, more comprehensive data from larger study samples are still lacking. Therefore, the present analysis sought to provide data on the safety pharmacology of single-dose administrations of LSD. We primarily addressed acute subjective, physiologic, and adverse effects during the LSD response (0–12 h after administration) and subacute adverse effects up to 24 h after administration. We also included data on adverse events that occurred during the entire clinical studies, even potentially days after LSD administration, and blood laboratory markers of kidney and liver function at both the start and end of the study. These data were collected from a series of clinical Phase 1 trials in healthy subjects that were conducted in the same laboratory and used the same standardized data recording methods (Holze et al. 2020, 2021a; Schmid et al. 2015; Dolder et al. 2016, 2017), thereby facilitating pooling of the data. The studies used a dose range of LSD base from low (25 µg) to moderate (50 µg) to moderate-high (100 µg) to high (200 µg) experiential doses as used in LSD-assisted psychotherapy (Gasser et al. 2015, 2014; Schmid et al. 2021) and in subjects with no or minimal prior LSD use, which is also likely the case when LSD is used in patients.

## Methods

### Study design

This was a pooled analysis of four double-blind, placebo-controlled, random-order, crossover studies in healthy subjects (Schmid et al. 2015; Holze et al. 2021b, 2020, 2019; Dolder et al. 2015, 2016). These studies were all conducted at the University Hospital Basel and included a total of 83 participants who were all psychiatrically screened and healthy. The aim of the pooled analysis was to assess the safety pharmacology of single doses of LSD in healthy subjects with no regular LSD use and no or minimal previous use. The first study (Study 1) included 16 healthy subjects who received a single administration of 200 µg LSD and placebo. The second study (Study 2) included 24 healthy subjects who received a single administration of 100 µg LSD and placebo. Studies 1 and 2 did not use pharmaceutically well-defined doses. Therefore, the doses were adjusted in the present analysis based on individual plasma LSD concentrations by taking data from Study 4 as a reference. Study 3 included 27 healthy subjects who received single doses of methylenedioxymethamphetamine (MDMA), D-amphetamine, 100 µg LSD, and placebo. Study 4 included 16 healthy subjects who received 25, 50, 100, and 200 µg LSD, 200 µg LSD + ketanserin (serotonin 5-hydroxytryptamine-2 receptor antagonist), and placebo. Only the LSD alone and placebo conditions were used for the present pooled analysis. Overall, all four studies resulted in a total of 131 LSD administrations. In all of the studies, the washout periods between single drug dose administrations were at least 10 days to exclude carry-over effects. The studies were all registered at ClinicalTrials.gov (NCT01878942, NCT02308969, NCT03019822, and NCT03321136) and approved by the local ethics committee. The studies were conducted in accordance with the Declaration of Helsinki. LSD administration in healthy subjects was authorized by the Swiss Federal Office for Public Health (BAG), Bern, Switzerland. Informed consent was obtained from all of the participants who were included in the studies. All of the subjects were paid for their participation.

### Subjects and dose groups

Characteristics of the study participants are shown in Table 1. A total of 83 healthy European/Caucasian subjects (41 men, 42 women), 25–60 years old (mean ± SD = 30 ± 8 years; range: 25–60 years), were mostly recruited from the University of Basel campus and included in the studies. The mean ± SD (range) ages were 33 ± 12 (25–60) years, 29 ± 6 (25–51) years, 28 ± 4 (25–45) years, and 29 ± 6 (25–52) years for Study 1, Study 2, Study 3, and Study 4, respectively. The mean ± SD body weight was 70 ± 12 kg (range: 50–98 kg). Sixteen participants received four single-dose administrations of LSD at different doses (25, 50, 100, and 200 µg), and 67 participants received a single dose of LSD only (either 100 or 200 µg).

**Table 1:** Demographics of study participants and assignment to dose group

Dose group		Placebo	“25 micg LSD”	“50 µg LSD”	“100 µg LSD”	“200 micg LSD”
Administrations	N	83	19	35	45	32
Group average of target doses	µg	0	37 ± 28	77 ± 25	111 ± 32	184 ± 37
Target dose range	µg	0	25–100	50–100	100–200	100–200
LSD C_max_ (Study 1–4)	pg/mL	0	540 ± 158	1224 ± 210	2022 ± 286	3921 ± 1089
Range LSD C_max_ (Study 1–4)	pg/mL	0	207–780	800–1537	1569–2529	2760–7350
Body weight	kg	70 ± 12	70 ± 14	69 ± 14	70 ± 12	70 ± 14
Participant age	years	30 ± 8	29 ± 7	31 ± 9	29 ± 7	28 ± 5
Range participant age	years	25–60	25–52	25–60	25–53	25–52
Administrations Study 1	N	16	0	0	4	12
Administrations Study 2	N	24	3	11	8	2
Administrations Study 3	N	27	0	8	17	2
Administrations Study 4	N	16	16	16	16	16
Reference LSD C_max_ from Study 4	pg/mL	0	510	1100	2000	3800
Range reference LSD C_max_ from Study 4	pg/mL	0	210–690	790–1500	1600–2900	2400–6900

Target dose, the dose unit (25, 50, 100, or 200 µg administered to the participant); *N*, number of subjects; data are mean ± SD unless indicated otherwise

Dose groups for Studies 1–3 were adjusted based on individual maximal concentrations of LSD in plasma. The plasma concentrations in Study 4 served as a reference for grouping because the administered doses of LSD were pharmaceutically exactly defined, including content unity and stability of the formulation of all dose strengths, whereas this was not the case for the formulations that were used in Studies 1 and 2. We believe that some of the capsules that were used in Studies 1 and 2 contained less LSD than indicated/targeted mainly because of inactivation to iso-LSD over time (Steuer et al. 2017; Holze et al. 2021a). Full plasma LSD concentration–time curves were available for all participants. LSD peak plasma levels were used to newly assign the participants to dose groups based on their actual exposure to LSD rather than the targeted and unknown dose of LSD that was used (Table 1). Based on plasma LSD levels, four of the 16 participants in Study 1 (targeted 200 µg) were newly assigned to the 100 µg group. For Study 2 (targeted 100 µg), three, 11, and two of the 24 participants were assigned to the 25, 50, and 200 µg dose groups, respectively. For Study 3 (targeted 100 µg), eight and two of the 27 participants were assigned to the 50 and 200 µg dose groups, respectively, although a novel LSD formulation was used in Study 3. The reference LSD peak concentrations were taken from Study 4 (Holze et al. 2021b) and are listed in Table 1. The actual peak concentrations over all studies (Studies 1–4) after regrouping are shown in Table 1.

Exclusion criteria were reported in detail elsewhere (Schmid et al. 2015; Holze et al. 2021b, 2020, 2019; Dolder et al. 2015, 2016) and included a history of psychiatric disorders, physical illness, a lifetime history of using illicit drugs more than 10 times (with the exception of past cannabis use), illicit drug use within the last 2 months, and illicit drug use during the study, determined by urine tests that were conducted before the test sessions. Forty-nine subjects had prior drug experience (1–10 times), of which 23 subjects had previously used a psychedelic (1–4 times). Further substance experiences included MDMA (35 subjects, 1–8 times), amphetamine (17 subjects, 1–3 times), cocaine (9 subjects, 1–4 times), methylphenidate (16 subjects, 1–3 times), and opium (1 subject, once).

### Study drug

Studies 1 and 2 used gelatin capsules that contained 100 µg of pharmaceutically pure LSD (D-lysergic acid diethylamide hydrate; Lipomed AG, Arlesheim, Switzerland). Corresponding placebo capsules were prepared by Bichsel Laboratories Interlaken (Interlaken, Switzerland). Quality control was performed by R. Brenneisen at the Department of Clinical Research, University of Bern, Switzerland, but stability of the formulation was not tested repeatedly or beyond the study completion date. Study 3 used an ethanolic LSD solution (D-lysergic acid diethylamide base, high-performance liquid chromatography purity > 99%, Lipomed AG, Arlesheim, Switzerland). The exact analytically confirmed LSD content (mean ± SD) of the formulation was 96.2 ± 0.3 µg after production. Study 4 used an identical formulation as Study 3, containing 25 or 100 µg LSD with an exact content of 25.7 ± 0.57 µg and 98.7 ± 1.6 µg, respectively. For Studies 3 and 4, stability of the formulation was confirmed repeatedly during and after study completion.

### Study procedures

All of the studies included a screening visit, two to six test sessions (each separated by at least 10 days), and an end-of-study visit. The sessions were conducted in a calm standard hospital room equipped with a standard hospital bed for the participant and a desk and a chair for the investigator. The room had an adjoining balcony, which participants were allowed to access after peak effects had subsided in company of the investigator. Only one research subject and one investigator were present during each test session. Participants were allowed to bring their own music and to bring occupation for the time after effects had subsided or for placebo days (e.g., book, laptop, games). Blindfolds were provided upon request. The test sessions began at approximately 8:00 AM. To ensure that the participants were prepared for the LSD-induced experience, individual emotional states were assessed before drug administration to exclude risk factors for emotional disturbances. This procedure consisted of several questions, including “Did anything unusual happen lately?,” “Do you feel stressed for any reason (personal or professional)?,” “Did you have any sleep disturbances lately?,” “Do you have any expectations or fear regarding today’s session?,” and “Are you feeling ready to participate today?” If any of these questions were answered with “yes” (or “no” for the last question), then the reason was discussed. If the investigator had any doubt, then the session was rescheduled to ensure that none of the participants was in an unfavorable state of mind when taking LSD. The subjects then underwent baseline measurements, including vital signs, to ensure basic physical health. LSD or placebo was administered at approximately 9:00 AM. The subjects were never alone during the next 12–16 h after drug administration, and the investigator was in a room next to the subject for up to 24 h (except for Study 3, in which subjects were sent home after 12 h, accompanied by a friend or family member).

### Pharmacodynamic measures

Visual Analog Scales (VASs) were repeatedly used to assess subjective effects over time (Hysek et al. 2014). The VASs included “any drug effect,” “good drug effect,” “bad drug effect,” and “anxiety.” The VASs were presented as 100-mm horizontal lines (0–100%), marked from “not at all” on the left to “extremely” on the right. The VASs were applied before and 0, 0.5, 1, 1.5, 2, 2.5, 3, 4, 5, 6, 7, 8, 9, 10, 11, 12, 14, 16, and 24 h after LSD or placebo administration. In Study 1, the 14-h time point was not used. In Study 2, the 2.5- and 14-h time points were not used. In Study 3, VASs were assessed before and 0, 0.5, 1, 1.5, 2, 3, 3.5, 4.5, 5.5, 6.5, 7.5, 8.5, 9.5, 10.5, and 11.5 h after administration. Severe anxiety was defined as > 75% on the “anxiety” VAS. The onset, offset, and duration of the subjective response were determined using the “any drug effect” VAS-time curve, with 10% of the individual maximal response as the threshold, in Phoenix WinNonlin 6.4 (Pharsight, Certara L.P., St. Louis, MO, USA). The 5 Dimensions of Altered States of Consciousness (5D-ASC) scale (Dittrich 1998; Studerus et al. 2010) was administered 24 h (or 11.5 h in Study 3) after drug administration to retrospectively rate peak drug effects. The “Oceanic Boundlessness” (OB) and “Anxious Ego-Dissolution” (AED) dimensions are reported to serve as approximation to describe rather positive and negative alterations of mind that were induced by LSD, respectively (Holze et al. 2021b; Liechti et al. 2017).

Blood pressure, heart rate, and body temperature were assessed repeatedly at the same time points when the VASs were administered. Systolic and diastolic blood pressure and heart rate were measured using an automatic oscillometric device (OMRON Healthcare Europe NA, Hoofddorp, Netherlands). The measurements were performed in duplicate at an interval of 1 min and after a resting time of at least 10 min. Averages were used for further analysis. Core (tympanic) temperature was measured using a Braun ThermoScan ear thermometer (Welch Allyn, Skaneateles Falls, NY, USA). Criteria for grouping subjects into proportions with a certain degree of stimulation were diastolic blood pressure > 90, > 100, and > 110 mmHg and systolic blood pressure > 140, > 160, and > 180 mmHg. Tachycardia was defined as > 100 beats/min. Hyperthermia and hyperpyrexia were defined as tympanic body temperature > 38 °C and 40 °C, respectively.

Acute and subacute adverse effects were assessed using the List of Complaints ((Zerssen 1976; Hysek et al. 2012a, b). The scale consists of 66 items, yielding a total adverse effects score (non-weighted sum of item answers) that reliably measures physical and general discomfort. The List of Complaints was administered before and 10–12 h (acute adverse effects up to 12 h) and 24 h (subacute adverse effects up to 24 h) after LSD or placebo administration. Subacute adverse effects were not recorded in Study 3. Additionally, participants were asked at the beginning of each study session and at the end of study visit to report any adverse events from 24 h after drug administration until the next study visit. Adverse events were assessed in consultation with a study physician.

### Blood sampling and end-of-study visit

Blood chemistry and blood cell count tests were performed at the screening visit at the start of the study and at the end-of-study visit, which were separated by 107 ± 63 days (mean ± SD). The end-of-study visit, including blood sampling, occurred at variable time intervals (28 ± 21 days) after the last LSD administration. The analyses were performed using standard assays according to Good Laboratory Practice by the Laboratory Medicine Department of the hospital. The glomerular filtration rate was determined by the Cockcroft-Gault Equation using plasma creatinine concentrations, age, and sex of the subject. At the end-of-study visit, the participants were asked to retrospectively rate whether the experience was positive or negative, whether the controlled clinical setting influenced their experience, and whether they considered taking LSD again and in what setting. The participants were also asked whether they experienced “flashbacks” or any other change in perception (e.g., altered spatial perception, vision of color or patterns) and for how long they lasted. “Flashbacks” were defined as temporary reoccurrence of the altered state of consciousness, whereas persistent changes in perception would have led to further assessments in regard to HPPD. This was assessed in a structured manner only at the end-of-study visit and therefore we only report “flashback” phenomena that occurred until the end-of-study visit. “Flashbacks” that occurred outside this period of time were not assessed.

### Statistical analyses

The statistical analyses were performed using Statistica 12 software (StatSoft, Tulsa, OK, USA). Analyses of variance (ANOVAs), with drug as the within-subjects factor and dose as the between-subjects factor, were used to evaluate all of the effects of LSD compared with placebo (main effect of drug) and dose–response effects (drug × dose interactions). The significant main effects or interactions in the ANOVA were followed by Tukey’s post hoc test. Fisher’s exact tests were used to compare proportions. Differences in kidney and liver function and blood cell counts between the screening and end-of-study visit measures were analyzed using paired *t*-tests. The level of significance was set to *p* < 0.05.

## Results

### Acute subjective effects of LSD

“Any drug effect” and “good drug effect” on the VAS and OB ratings on the 5D-ASC scale dose-dependently increased up to 100 µg LSD, with a ceiling effect at 100 µg. Negative effects, including “bad drug effect” and “anxiety,” on the VAS and AED ratings on the 5D-ASC dose-dependently increased up to 200 µg LSD, with no ceiling effect (Table 2). Positive subjective drug effects, including “good drug effect,” on the VAS and OB ratings on the 5D-ASC also showed steeper dose–effect curves and higher maximal effects compared with negative subjective drug effects, including “bad drug effect” and anxiety ratings on the VAS and AED ratings on the 5D-ASC scale, respectively (Table 2). The effect duration (mean ± SD) was dose dependent and 4.5 ± 2.4 h, 7.2 ± 2.5 h, 8.5 ± 3.2 h, and 11 ± 4.6 h for the 25, 50, 100, and 200 µg LSD doses, respectively. Time to onset was 1.2 ± 0.6 h, 0.7 ± 0.4 h, 0.5 ± 0.3 h, and 0.4 ± 0.4 h, and the time to peak effect was 2.9 ± 1.1 h, 2.8 ± 1.1 h, 2.5 ± 1.1 h, and 2.0 ± 1.2 h, for the 25, 50, 100, and 200 µg LSD doses, respectively.

**Table 2:** Subjective and adverse effects of LSD in healthy subjects

Emax (mean ± SD)								
**LSD dose**		**Placebo** **(*N* = 83)**	**“25 µg”** **(*N* = 19)**	**“50 µg”** **(*N* = 35)**	**“100 µg”** **(*N* = 45)**	**“200 µg”** **(*N* = 32)**	***F***_**4,209**_	***P* = **
**Visual Analog Scales (VAS)**								
Any drug effect		1.0 ± 3.6	30 ± 28***	72 ± 24***###	89 ± 16***### + + +	88 ± 24***### + +	280	< 0.001***
> 25, *N* (%)		0 (0)	9 (47)***	33 (94)***###	45 (100)***###	30 (94)***###		
> 50, *N* (%)		0 (0)	4 (21)***	28 (80)***###	44 (98)***### +	29 (91)***###		
> 75, *N* (%)		0 (0)	2 (11)*	18 (51)***##	36 (80)***### + +	28 (88)***### + +		
100, *N* (%)		0 (0)	1 (5)	7 (20)***	21 (47)***## +	17 (53)***### + +		
	Good drug effect	1.5 ± 7.8	38 ± 29***	73 ± 25***###	86 ± 18***### +	86 ± 26***### +	215	< 0.001***
> 25, *N* (%)		1 (1)	12 (63)***	33 (94)***##	45 (100)***###	30 (94)***##		
> 50, *N* (%)		1 (1)	7 (37)***	32 (91)***###	43 (96)***###	29 (91)***###		
> 75, *N* (%)		0 (0)	3 (16)**	19 (54)***##	34 (76)***###	26 (81)***### +		
	Bad drug effect	0.1 ± 0.3	2.8 ± 5.6	14 ± 19**	24 ± 27***###	32 ± 33***### + +	21	< 0.001***
> 25, *N* (%)		0 (0)	0 (0)	7 (20)***#	17 (38)***##	13 (41)***###		
> 50, *N* (%)		0 (0)	0 (0)	3 (9)*	12 (27)***# +	10 (31)***## +		
> 75, *N* (%)		0 (0)	0 (0)	0 (0)	3 (7)*	4 (13)** +		
Anxiety		0.2 ± 1.1	0.2 ± 0.5	6.6 ± 12	14 ± 26**	25 ± 34***### + + +	12	< 0.001***
> 25, *N* (%)		0 (0)	0 (0)	3 (9)*	12 (27)***# +	10 (31)***## +		
> 50, *N* (%)		0 (0)	0 (0)	1 (3)	9 (20)***# +	9 (28)***# + +		
> 75, *N* (%)		0 (0)	0 (0)	0 (0)	3 (7)*	5 (16)** +		
**5-Dimensions of Altered States of Consciousness (5D-ASC) scale**								
Oceanic Boundlessness (OB)(%)		0.0 ± 0.1	10 ± 23	25 ± 20***#	41 ± 23***### + +	44 ± 25***### + +	58	< 0.001***
Anxious Ego-Dissolution (AED)(%)		0 ± 0	3.4 ± 8.8	13 ± 16***	20 ± 15***###	22 ± 23***### +	28	< 0.001***
**List of Complaints (LC) total score**								
Before, *N*		1.2 ± 2.8	0.8 ± 1.1	1.1 ± 1.4	1.3 ± 2.1	0.8 ± 1.2	0.4	NS
Acute adverse effects, up to 12 h, *N*		2.7 ± 4.4	5.6 ± 4.9	9.2 ± 7.6***	12 ± 11***#	13 ± 11***##	16	< 0.001***
Subacute adverse effects, up to 24 h, *N*		0.8 ± 1.5	2.7 ± 4.5	4.8 ± 6.4*	5.9 ± 7.7***	6.3 ± 7.3***	7.0	< 0.001***

^*^P < 0.05, **P < 0.01, ***P < 0.001 compared to placebo, #*P* < 0.05, ##*P* < 0.01, ###*P* < 0.001 compared to “25 µg”; + *P* < 0.05, +  + *P* < 0.01, +  +  + *P* < 0.001 compared to “50 µg”; data shown as mean ± SD if not indicated otherwise; *NS*, not significant; *N*, number of subjects; *SD*, standard deviation

### Acute effects of LSD on vital signs

LSD produced significant acute and transient increases in blood pressure, heart rate, and body temperature at doses > 25 µg (Table 3). A dose-dependent effect was observed for elevations of heart rate and body temperature but not blood pressure (Table 3). Systolic blood pressure > 140, > 160, and > 180 mmHg was observed in 48%, 5%, and 0% of all LSD administrations, respectively. Maximal diastolic and systolic blood pressure values among the 131 LSD administrations were 103 and 173 mmHg, respectively. Tachycardia was observed in 15% of all LSD administrations, and the highest heart rate of any subject was 129 beats/min. LSD increased body temperature to > 38 °C in 14% of all LSD administrations. The highest body temperature was 38.8 °C. No hyperpyrexia (> 40 °C) occurred.

**Table 3:** Maximal effects of LSD on vital signs

Dose							
**LSD dose**	**Placebo** **(*N* = 83)**	**“25 µg”** **(*N* = 19)**	**“50 µg”** **(*N* = 35)**	**“100 µg”** **(*N* = 45)**	**“200 µg”** **(*N* = 32)**	***F***_**4,209**_	***P* = **
Diastolic blood pressure (mean ± SD, mmHg)	78 ± 7	84 ± 7*	86 ± 8**	87 ± 8***	86 ± 7***	13.5	< 0.001***
> 90, *N* (%)	5 (6)	3 (15)	12 (34)***	19 (42)***#	9 (28)**		
> 100, *N* (%)	0 (0)	0 (0)	0 (0)	2 (4)	0 (0)		
Max, mmHg	99	99	100	103	100		
Systolic blood pressure (mean ± SD, mmHg)	129 ± 12	134 ± 12	139 ± 13**	141 ± 13***	142 ± 11***	10	< 0.001***
> 140, *N* (%)	17 (20)	4 (21)	16 (46)**	24 (53)***#	19 (59)***##		
> 160, *N* (%)	1 (1)	0 (0)	1 (3)	4 (9)	2 (6)		
> 180, *N* (%)	0 (0)	0 (0)	0 (0)	0 (0)	0 (0)		
Max, mmHg	161	156	172	173	170		
Heart rate (mean ± SD, beats/min)	73 ± 10	75 ± 11	80 ± 14*	86 ± 15***#	90 ± 14***## +	13.1	< 0.001***
> 80, *N* (%)	23 (28)	7 (37)	16 (46)	27 (68)***	22 (69)***#		
> 100, *N* (%)	0 (0)	0 (0)	2 (6)	9 (20)***#	8 (25)***# +		
> 120, *N* (%)	0 (0)	0 (0)	1 (3)	0 (0)	1 (3)		
Max, beats/min	98	94	129	118	121		
Body temperature (mean ± SD, °C)	37.3 ± 0.4	37.3 ± 0.3	37.5 ± 0.3*	37.6 ± 0.3***#	37.8 ± 0.5***### +	11.8	< 0.001***
> 38, *N* (%)	3 (4)	0 (0)	4 (11)	3 (7)	11 (34)***## + ‡‡		
Max, °C	38.8	37.8	38.2	38.4	38.8		

*N*, number of subjects; *SD*, standard deviation; *P < 0.05, **P < 0.01, ***P < 0.001 compared to placebo, #*P* < 0.05, ##*P* < 0.01, ###*P* < 0.001 compared to 25 µg; + *P* < 0.05, compared to 50 µg; ‡‡*P* < 0.01, compared to 100 µg

### Adverse effects of LSD

LSD produced significant acute and subacute adverse effects on the List of Complaints (LC) compared with placebo (Table 2). Adverse effects were comparable at the 50, 100, and 200 µg doses and greater than placebo and the 25 µg dose of LSD (Table 2). Specific complaints are listed in Table 4. The most frequent acute adverse effects after LSD administration listed on the LC included lack of concentration, lack of appetite, feeling of physical or emotional weakness, restlessness, impaired balance, headache, forgetfulness, dizziness, brooding, perspiration, and hypersensitivity to certain odors (Table 4). Additional possibly treatment-related adverse events that were spontaneously reported within the first 48 h after discharge from the study visits included headache (5% after LSD, 10% after placebo), migraine (5% after LSD, 1% after placebo), common cold (7% after LSD, 10% after placebo), gastroenteritis (1% after LSD, 0% after placebo), flatulence (1% after LSD, 0% after placebo), diarrhea (4% after LSD, 0% after placebo), and insomnia (1% after LSD, 0% after placebo). Six subjects (7%) reported flashbacks 1–3 times after LSD administration. Flashbacks reportedly occurred 43 ± 11 h (mean ± SD) after LSD administration (range: 24–86 h). None of the subjects reported persisting perceptual alterations. No serious adverse reactions occurred.

**Table 4:** Acute and subacute adverse effects of LSD on the List of Complaints (LC)

Acute adverse effects (up to 12 h)						Subacute adverse effects (up to 24 h)				
**LSD dose**	**Placebo** **(*N* = 83)**	**“25 µg”** **(*N* = 19)**	**“50 µg”** **(*N* = 35)**	**“100 µg”** **(*N* = 44)**	**“200 µg”** **(*N* = 31)**	**Placebo** **(*N* = 56)**	**“25 µg”** **(*N* = 19)**	**“50 µg”** **(*N* = 27)**	**“100 µg”** **(*N* = 28)**	**“200 µg”** **(*N* = 29)**
***N* (%)**										
Lack of concentration	7 (8)	8 (42)**	15 (43)***	28 (64)***	22 (71)*** +	1 (2)	1 (5)	1 (4)	6 (21)**	5 (17)*
Lack of appetite	7 (8)	4 (21)	11 (31)**	24 (55)***# +	16 (52)***	0 (0)	0 (0)	0 (0)	3 (11)*	4 (14)*
Subjective feeling of weakness	1 (1)	3 (16)*	9 (26)***	20 (45)***#	12 (39)***#	0 (0)	0 (0)	2 (7)	4 (14)*	7 (24)***#
Restlessness	1 (1)	1 (5)	9 (26)***	17 (39)***##	11 (35)***#	0 (0)	0 (0)	1 (4)	2 (7)	3 (10)*
Impaired balance	1 (1)	2 (11)	6 (17)**	16 (36)***	13 (42)***# +	0 (0)	0 (0)	1 (4)	1 (4)	2 (7)
Headache	16 (19)	7 (37)	13 (37)	23 (52)***	16 (52)**	11 (20)	4 (21)	6 (22)	12 (43)*	12 (41)*
Forgetfulness	1 (1)	2 (11)	5 (14)**	13 (30)***	13 (42)***# +	0 (0)	0 (0)	2 (7)	4 (14)	5 (17)**
Dizziness	0 (0)	4 (21)***	3 (9)*	15 (34)*** + +	9 (29)***	0 (0)	0 (0)	1 (4)	2 (7)	2 (7)
Brooding	3 (4)	2 (11)	5 (14)*	16 (36)*** +	11 (35)***	0 (0)	0 (0)	1 (4)	5 (18)**	6 (21)**
Perspiration	5 (6)	3 (16)	5 (14)	18 (41)*** +	10 (32)***	0 (0)	0 (0)	2 (7)	2 (7)	2 (7)
Hypersensitivity to certain odors	1 (1)	2 (11)	10 (29)***	8 (18)***	9 (29)***	0 (0)	0 (0)	2 (7)	2 (7)	2 (7)
Feeling exhausted	4 (5)	0 (0)	5 (14)	13 (30)***## + +	14 (45)***### + +	1 (2)	0 (0)	1 (4)	3 (11)	5 (17)*
Feeling dull	8 (10)	5 (26)	6 (17)	15 (34)**	11 (35)**	4 (7)	1 (5)	2 (7)	7 (25)*	11 (38)***#
Flushing	3 (4)	1 (5)	7 (20)**	15 (34)***#	6 (19)*	0 (0)	1 (5)	2 (7)	3 (11)*	0 (0)
Nausea	7 (8)	3 (16)	6 (17)	15 (34)***	11 (35)**	2 (4)	0 (0)	0 (0)	1 (4)	3 (10)
Lack of energy	7 (8)	3 (16)	5 (14)	16 (36)*** +	11 (35)**	2 (4)	0 (0)	1 (4)	5 (18)*	7 (24)**#
Bruxism	6 (7)	0 (0)	6 (17)	19 (43)***### +	8 (26)*#	1 (2)	0 (0)	1 (4)	1 (4)*	1 (3)
Restless legs	1 (1)	2 (11)	5 (14)**	11 (25)***	7 (23)***	0 (0)	1 (5)	0 (0)	2 (7)	1 (3)
Shivering	3 (4)	1 (5)	3 (9)	11 (25)***	10 (32)***# +	0 (0)	0 (0)	1 (4)	1 (4)	1 (3)
Agitation	4 (5)	0 (0)	4 (11)	14 (32)***##	8 (26)**#	0 (0)	0 (0)	0 (0)	3 (11)*	3 (10)*
Abdominal pain	3 (4)	3 (16)	7 (20)**	9 (20)**	5 (16)*	0 (0)	0 (0)	2 (7)	1 (4)	2 (7)
Heavy legs	4 (5)	2 (11)	4 (11)	11 (25)**	8 (26)**	1 (2)	1 (5)	1 (4)	2 (7)	3 (10)
Not feeling at ease	3 (4)	0 (0)	2 (6)	12 (27)***# +	6 (19)*	0 (0)	0 (0)	1 (4)	2 (7)	1 (3)
Strong thirst	10 (12)	2 (11)	6 (17)	14 (32)**	9 (29)*	2 (4)	2 (11)	3 (11)	3 (11)	4 (14)
Feeling of pressure or abdominal fullness	5 (4)	0 (0)	4 (11)	10 (23)**#	9 (29)**##	0 (0)	0 (0)	0 (0)	2 (7)	2 (7)
Anxiety	2 (6)	0 (0)	1 (3)	12 (27)***#	5 (16)*	0 (0)	0 (0)	0 (0)	2 (7)	2 (7)
Urge to urinate	5 (6)	2 (11)	6 (17)	8 (18)	6 (19)	1 (2)	1 (5)	1 (4)	1 (4)	3 (10)
Tiredness	28 (34)	4 (21)	14 (40)	24 (55)*#	16 (52)#	14 (25)	8 (42)	10 (37)	15 (54)*	16 (55)**
Freezing	5 (6)	1 (5)	0 (0)	10 (23)** + +	2 (6)	0 (0)	0 (0)	0 (0)	1 (4)	1 (3)

*N*, number of subjects; *P < 0.05, **P < 0.01, ***P < 0.001 compared with placebo; #*P* < 0.05, ##*P* < 0.01, ###*P* < 0.001 compared with “25 µg”; + *P* < 0.05, +  + *P* < 0.01 compared with “50 µg” (Fisher’s exact test)

### Plasma LSD concentrations

Plasma peak concentrations of LSD for all dose groups are shown in Table 1. The full pharmacokinetics of LSD that were used in the studies are reported in detail elsewhere (Holze et al. 2021b, 2019; Dolder et al. 2017).

### Effects of LSD on kidney and liver function and changes in blood cell counts

At the end of the study and 28 ± 21 days (mean ± SD) after the last administration of LSD, plasma creatinine levels and the estimated glomerular filtration rate were unchanged compared with the start of the study and before LSD administration (Table 5). Similarly, plasma levels of alanine aminotransferase and γ-glutamyl transpeptidase were similar at the screening visit and end-of-study visit. Red blood cell counts and hemoglobin levels decreased, and thrombocyte levels increased during the studies. White blood cell counts remained unchanged.

**Table 5:** Kidney and liver function parameters and blood cell counts before and at study end

Screening		End of study	*t*-test	
**Kidney and liver function**	***N* = 81**^**a**^		***t***	***P* = **
Creatinine (normal: < 97 µM)				
Mean ± SD, µM (range)	73 ± 13 (54–106)	72 ± 12 (53–108)	0.94	NS
Glomerular filtration rate C_CR_ (normal: > 90 ml/min)				
Mean ± SD, ml/min (range)	122 ± 27 (59–236)	123 ± 27 (56–221)	− 0.69	NS
Alanine aminotransferase (normal: < 59 U/I)				
Mean ± SD, U/l (range)	20 ± 7 (10–44)	20 ± 10 (8–62)	− 0.41	NS
**Blood cell counts**	***N* = 81**^**a**^			
White blood cells (normal: 3.5–10.0 × 10^9^/l)				
Mean ± SD, × 10^9^/l (range)	6.4 ± 1.5 (3.9–10.0)	6.2 ± 1.5 (3.6–12.4)	0.79	NS
Red blood cells (normal: 4.2–6.3 × 10^12^/l)				
Mean ± SD, × 10^12^/l (range)	4.7 ± 0.4 (3.9–5.5)	4.6 ± 0.4 (3.6–5.7)	2.13	< 0.05
Hemoglobin (normal: 120–180 g/l)				
Mean ± SD, g/l (range)	145 ± 12 (121–171)	141 ± 14 (114–176)	2.89	< 0.05
Thrombocytes (normal: 150–450 × 10^9^/l)				
Mean ± SD, × 10^9^/l (range)	234 ± 56 (93–375)	245 ± 70 (49–441)	− 2.22	< 0.05

*SD*, standard deviation; *N*, number of subjects; a data from 2 subjects missing

### Subjects’ interest in using LSD again

Seventy-five percent of the subjects were LSD-naive at the time of the studies, and the other 25% had very limited experience with LSD (i.e., maximum ≤ 3 exposures). Eighty-three subjects were asked whether they would consider taking LSD again. Three subjects (2%) reported that they would probably not take LSD again under any circumstances. Fifty-seven subjects (69%) reported that they would consider taking LSD again. Twenty-nine subjects (35%) reported that they might consider taking LSD again. Twenty-three subjects (29%) would not take LSD in a recreational setting but might consider participating in another study that administers LSD under controlled conditions. Twenty-six subjects (33%) indicated that they might consider taking LSD in a recreational setting but only in a protected environment. Thirteen of these 26 subjects (50%) had taken illicit drugs previously, and seven of these latter 13 (50%) had a previous experience with a psychedelic. Sixty subjects (72%) reported a positive overall LSD experience, 16 subjects (19%) reported a neutral experience, and seven subjects (8%) reported a disappointing or bad experience. No sex differences were observed. Sixteen subjects (19%) reported that the controlled setting had no impact on their experience, whereas 66 subjects (80%) reported that the controlled setting was important for their type of experience and was reassuring and made them feel safe. One participant (1%) reported that the setting was suboptimal.

## Discussion

The present analysis pooled data from four placebo-controlled studies of LSD and mainly characterized acute subjective, physiologic, and adverse effects of different doses in healthy subjects. The acute subjective effects of LSD were well tolerated in the studies that were included in the present analysis. LSD produced dose-dependent subjective good drug effects in most participants, with “good drug effect” ratings that were higher than 75% of the scale maximum in 76% and 81% of the participants at the 100 and 200 µg LSD doses, respectively. Subjective “bad drug effect” ratings were comparatively low, with ratings > 75% in 7% and 13% of the participants at the 100 and 200 µg LSD doses, respectively, and lasting no longer than 6 and 8 h for the 100 and 200 µg LSD doses, respectively. Overall, positive subjective drug effects, including “good drug effect” and OB, were reached at lower doses and to a higher extent than negative subjective drug effects, including “bad drug effect,” “anxiety,” and AED. The present findings indicate a therapeutic dose window for the induction of positive over negative subjective acute responses to LSD in most subjects. Elevations of blood pressure, heart rate, and body temperature were moderate at all doses of LSD that were used in the present analysis, indicating that LSD has only moderate cardiostimulant effects in healthy subjects. LSD also produced acute and subacute adverse effects, including feelings of tiredness, headache, impaired balance, and lack of concentration. However, these untoward effects are not severe, with no serious adverse reactions. The present analysis is the largest and most comprehensive analysis of the acute safety of LSD in healthy subjects. The present findings are consistent with previously published studies that include subsets from this pooled analysis (Dolder et al. 2016; Schmid et al. 2015; Holze et al. 2021b, 2020). The present analysis confirms and extends a previous, smaller crossover dose–response study that provided the plasma concentration reference groups for this study (Holze et al. 2021b). In contrast to the previous studies’ contributing data, the present pooled analysis focused on reporting proportions of participants who reached extreme values and potentially less frequent toxicity rather than only population means.

The risks of acute LSD administration are more psychological in nature rather than physiological (Johnson et al. 2008). The goal of LSD administration is to induce a mostly positive experience without anxiety, which is also predictive of a positive long-term therapeutic outcome in patients with depression, anxiety, or anxiety related to a terminal illness (Roseman et al. 2017; Ross et al. 2016; Griffiths et al. 2016) and long-term positive mood effects in healthy subjects (Schmid and Liechti 2018). In the present analysis, anxiety was assessed using different measures. Anxiety was reported on the List of Complaints as an acute adverse effect in 14% of all LSD administrations and not with the 25 µg dose. The “anxiety” VAS was assessed repeatedly, the ratings of which dose-dependently increased. Strong “anxiety” (> 75%) was reported in 6% of LSD administrations and only at the 100 and 200 µg LSD doses. During the study sessions, anxiety could be reduced by verbal support in all of the subjects, and benzodiazepines were not used. No cases of severe anxiety, panic attacks, or acute suicidality occurred. Overall, LSD induced predominantly positive experiences, reflected by nominally higher ratings of “good drug effect” and OB relative to “bad drug effect,” “anxiety,” and AED. Presumably, undesired negative subjective drug effects are likely more frequent in non-therapeutic settings and vulnerable individuals (Barrett et al. 2017). Negative experiences (so-called bad trips) that are induced by full doses of psychedelics, including severe anxiety, negative feelings, or panic attacks, are sometimes referred to as “challenging experiences” to indicate that these experiences may have some therapeutic value (Barrett et al. 2016). However, research in patients with depression shows that the acute experience predicts therapeutic outcome. High ratings of OB and low ratings of AED after psilocybin administration correlated with positive therapeutic outcomes in patients with treatment-resistant depression (Roseman et al. 2017). Additionally, a number of “set and setting” factors modulate the acute response to psychedelics, personality traits, mood states, and environment (Studerus et al. 2012). However, the most important determinant of the acute response to a psychoactive substance appears to be dose or plasma concentration of the active substance (Holze et al. 2021b, 2021a; Studerus et al. 2012; Hirschfeld and Schmidt 2021). Nevertheless, these factors remain to be better investigated in modern studies of LSD.

Retrospectively, over 70% of the participants in this pooled analysis reported overall positive subjective experiences, whereas only 8% of the subjects were disappointed by the effects of LSD or had bad experiences. Interestingly, the retrospective ratings of positive experiences were slightly higher than in a similar study that investigated the safety of MDMA, which has been shown to produce predominantly positive mood across laboratories (Vizeli and Liechti 2017; Baylen and Rosenberg 2006; Bershad et al. 2019a; Hernandez-Lopez et al. 2002). These findings on the subjective perception of the psychedelic experience indicate that more positive than negative experiences occurred under controlled conditions.

The importance of “set and setting” has previously been highlighted by many researchers using psychedelics (Johnson et al. 2008; Barrett et al. 2017; Nichols 2016; Hartogsohn 2016; Carbonaro et al. 2016). “Set” refers to the participants’ personality and the state of mind in which participants find themselves before the experience. To ensure that the participants were ready for the experience, individual emotional states were assessed immediately before LSD administration to exclude possible risk factors for emotional disturbances. “Setting” refers to the environment where sessions occur. The environment mostly needs to provide an individual feeling of security (Johnson et al. 2008). All of the studies were conducted in a standard quiet hospital room with only one investigator continuously present. The subjective response to LSD may be different in patients with psychiatric disorders compared with subjects who are screened to be psychiatrically healthy. Additionally, there are several concepts of a therapeutic “setting.” Some therapeutic studies of psychedelics used a one-to-one setting, but other substance-assisted therapies are conducted in a group setting that may also influence the subjective experience (Schmid et al. 2021; Gasser 2017).

The present analysis also determined the time course of the subjective response to LSD for all doses. The onset times, peak times, and effect durations were dose dependent. Effect durations increased with increasing doses, whereas onset and peak times decreased with increasing doses. The longer effect duration with higher doses is consistent with the close LSD-plasma concentration-effect relationship over time within subjects and a longer presence of LSD at the 5-HT_2A_ receptor (Holze et al. 2021b, 2019). These findings are consistent with a previous study that had a smaller sample size and that included subsets from this pooled analysis (Holze et al. 2021b).

With regard to potential physical harm, LSD dose-dependently induced mild sympathomimetic activation. LSD produced mild hypertension (systolic blood pressure > 140 mmHg and/or diastolic blood pressure > 90 mmHg) in approximately 50% of all LSD administrations and moderate hypertension (systolic blood pressure > 160 mmHg and/or diastolic blood pressure > 100 mmHg) in approximately 5% of all LSD administrations. Furthermore, LSD-induced tachycardia (> 100 beats/min) in approximately 15% of all LSD administrations. No severe hypertension (systolic blood pressure > 180 mmHg) was observed. The structurally similar psychedelic psilocybin produced comparable sympathomimetic activation at 10, 20, and 30 mg/70 kg (Carbonaro et al. 2018). MDMA produced greater increases in systolic blood pressure than LSD and more frequent tachycardia in one study (Vizeli and Liechti 2017). MDMA produced greater increases in systolic blood pressure than LSD and comparable overall cardiovascular stimulation to LSD in a study that directly compared the two substances in the same participants (Holze et al. 2020).

The present analysis also found that LSD dose-dependently increased body temperature. However, body temperatures did not increase above 38.8 °C, which was the same maximum temperature that was measured under placebo conditions. Compared with LSD, hyperpyrexia (> 40 °C) represents the most important life-threatening complication of recreational MDMA use (Wood et al. 2016; Liechti et al. 2005; Liechti 2014; Grunau et al. 2010; Halpern et al. 2011; Henry et al. 1992). In contrast to LSD which manly acts as direct serotonergic agonist, MDMA releases both serotonin and norepinephrine and its additional action on the adrenergic system may explain its greater sympathomimetic properties (Hysek et al. 2012a, b; Hysek et al. 2010; Liechti 2014).

In the present analysis, the participants reported a series of LSD-induced acute and subacute adverse effects. In line with the acute sympathomimetic and subjective effects, the frequency of adverse effects was only dose dependent up to 100 µg, but remained largely unchanged between 100 and 200 µg. The number of acute and subacute adverse events dose-dependently increased. Frequent acute adverse events included tiredness, headache, impaired balance, feeling of physical or emotional weakness, lack of appetite, restlessness, and forgetfulness. Frequent reported subacute adverse events included tiredness, headache, weakness, dullness, lack of energy, and lack of concentration. The nature of the reported subacute adverse effects indicates a state of “exhaustion” that might be comparable to feelings after intense brainwork or physical exertion. Between-session adverse events were equally frequent after LSD and placebo administration.

Hallucinogen persisting perception disorder (i.e., “flashbacks”) has previously been described following the use of LSD and other psychedelics. However, the frequency and nature of flashbacks and risk factors are still unidentified (Halpern et al. 2016; Martinotti et al. 2018). Participants in the studies that were included in the present analysis were asked at the end of the studies whether they experienced flashbacks or persisting changes in perception. Six subjects (7%) reported flashbacks but only within 24–86 h after LSD administration. None of the participants reported persisting changes in perception. Thus, our analysis found no evidence of persisting perceptual alterations after LSD administration in a controlled setting after doses up to 200 µg LSD.

In the present analysis, LSD did not influence levels of liver enzymes on average 1 month after LSD administration. But, an expected decrease in red blood cell counts and increase in thrombocytes was observed at the end-of-study visit. These findings are consistent with the regeneration of blood cell production that is caused by the overall blood loss of 400–600 ml that is attributable to blood sampling during the test sessions and similarly observed in other pharmacokinetic studies (Vizeli and Liechti 2017) or after blood donation.

In the present analysis, the dose groups were assigned according to peak plasma concentrations of LSD. Previous studies showed that plasma concentrations of LSD are strongly linked to subjective experiences within the same subject, with a clear dose–effect relationship that reaches a ceiling effect for “any drug effect” and “good drug effect” at 100 µg (Holze et al. 2021b). This relationship was preserved in the present analysis, although this was a pooled analysis of combined data from different participants and studies yet, including a subset of this study. This finding indicates that plasma concentrations of LSD are a key predictor of the effects of LSD.

The present safety data can partially be applied to the use of LSD in patients. The study participants typically had no or very little previous LSD experience, similar to most patients. Furthermore, LSD-assisted therapy is typically used sporadically 2–3 times and spaced several weeks apart along in addition to conventional non-substance-assisted psychotherapy (Schmid et al. 2021). However, LSD microdosing using more frequent administrations may have a different safety profile. Consistent with the present data, there are no reports to date of serious adverse reactions to LSD or similar serotonergic psychedelics in modern clinical studies (Andersen et al. 2021).

The present analysis has several limitations. First, the doses were not well-defined in two of the pooled studies because of the use of unstable formulations (Holze et al. 2019), and outcome data were reassigned to dose groups based on pharmacokinetics to account for this aspect. While providing a more valid concentration-effect relationship, this approach does not account for individual differences in the bioavailability or metabolism of LSD (Vizeli et al. 2021). For example, subjects who are poor metabolizers of cytochrome P450 2D6 may have higher plasma concentrations of LSD (Vizeli et al. 2021) than extensive metabolizers and may have been wrongly assigned to a higher dose group in the present analysis. Nevertheless, the present approach allowed us to include a larger study sample, and the findings were consistent with a smaller study with well-defined doses of LSD, including a subset of data reported here (Holze et al. 2021b). Furthermore, the study designs were heterogeneous in terms of single- or multiple-dose administration of LSD, and therefore, experiences in the study administering several doses might have been partially affected by previous experiences. Although previous substance use has not been shown to affect the acute subjective effects in a previous smaller study including partly the same data (Holze et al. 2021b). Additionally, we included only psychiatrically healthy subjects. Thus, the risks of using LSD in patients within a therapeutic setting may be different and also need to be investigated. Additionally, the participants were mostly young and physically healthy, but older patients or patients with cardiovascular risk factors may be treated with LSD. Furthermore, we only included 83 participants who received LSD a total of 131 times. This sample size is too small to detect infrequent (0.1–1%) or rare (< 0.1%) adverse events. Additionally, a long-term follow-up is missing.

The present analysis has strengths. We assessed safety aspects for a range of LSD doses (25–200 µg). All of the data were derived from randomized, double-blind, placebo-controlled studies that were conducted in the same laboratory. Furthermore, full LSD-plasma concentration–time profiles were available from all participants, thereby providing an objective measure of exposure to LSD and allowing comparisons with other studies that used different formulations. Specifically, both LSD base and tartrate salts are used in research. One microgram dose unit of LSD base that was used in the present analysis and by some researchers (Holze et al. 2021b, 2020, 2021a, 2019; Hutten et al. 2020; Carhart-Harris et al. 2016) corresponds to 1.46 or 1.23 µg of LSD 1:1 or 2:1 tartrate salt, respectively, as used by other researchers (Bershad et al. 2020, 2019b; Family et al. 2020; Yanakieva et al. 2019).

## Conclusion

Single-dose administrations of LSD up to 200 µg were safe in regard to acute psychological and physical harm in healthy subjects in a controlled clinical setting. LSD dose-dependently induced mild cardiovascular stimulation. Acute subjective effects were predominantly positive, but transient anxiety, fear, and bad drug effects occurred. These safety data do not raise any concerns about single infrequent LSD administration in a controlled clinical setting. However, risks and benefits of using LSD in a therapeutic setting need further study.
